# Kidney-specific transposon-mediated gene transfer *in vivo*

**DOI:** 10.1038/srep44904

**Published:** 2017-03-20

**Authors:** Lauren E. Woodard, Jizhong Cheng, Richard C. Welch, Felisha M. Williams, Wentian Luo, Leslie S. Gewin, Matthew H. Wilson

**Affiliations:** 1 Department of Veterans Affairs, Nashville, TN 37212 USA; 2 Department of Medicine, Vanderbilt University Medical Center, Nashville, TN 37232 USA; 3 Department of Medicine, Baylor College of Medicine, Houston, TX 77030, USA; 4 Department of Cell and Developmental Biology, Vanderbilt University Medical Center, Nashville, TN 37232 USA; 5 Department of Pharmacology, Vanderbilt University Medical Center, Nashville, TN 37232 USA; 6 Department of Veterans Affairs, Houston, TX 77030 USA

## Abstract

Methods enabling kidney-specific gene transfer in adult mice are needed to develop new therapies for kidney disease. We attempted kidney-specific gene transfer following hydrodynamic tail vein injection using the kidney-specific podocin and gamma-glutamyl transferase promoters, but found expression primarily in the liver. In order to achieve kidney-specific transgene expression, we tested direct hydrodynamic injection of a DNA solution into the renal pelvis and found that luciferase expression was strong in the kidney and absent from extra-renal tissues. We observed heterogeneous, low-level transfection of the collecting duct, proximal tubule, distal tubule, interstitial cells, and rarely glomerular cells following injection. To assess renal injury, we performed the renal pelvis injections on uninephrectomised mice and found that their blood urea nitrogen was elevated at two days post-transfer but resolved within two weeks. Although luciferase expression quickly decreased following renal pelvis injection, the use of the *piggyBac* transposon system improved long-term expression. Immunosuppression with cyclophosphamide stabilised luciferase expression, suggesting immune clearance of the transfected cells occurs in immunocompetent animals. Injection of a transposon expressing erythropoietin raised the haematocrit, indicating that the developed injection technique can elicit a biologic effect *in vivo*. Hydrodynamic renal pelvis injection enables transposon mediated-kidney specific gene transfer in adult mice.

Long-term phenotypic correction of an intrinsic kidney disease has not yet been achieved using gene therapy. Gene transfer to the kidney has proven difficult, even in animal models. Nonetheless, chronic kidney disease plagues 11.5% of the population contributing to morbidity and mortality^1^. Major gene transfer development efforts have not been focused on kidney disease. There has been some success with virally expressed transgenes in the kidney via adeno-associated virus^2^ or lentivirus^3^; however, these therapies have not been shown to be kidney-specific. Intra-arterial injection of AAV2-GFP virus in rats appeared kidney-specific based on histologic samples from several organs, but required renal artery injection, flushing, and extended clamping, which have not been translated to a mouse model^4^. Therefore, we sought to test new methods that did not use viruses and control the specificity of gene expression through physical means to develop a simple, reproducible method of introducing plasmid DNA into the kidneys of adult mice. We previously injected a doxycycline-inducible *piggyBac* system into the renal vein to express the antioxidant glutathione S-transferase A4 and the insulin-like growth factor-1 receptor, in both cases reversing many effects of the gene knockout in mice^5^,^6^. In this study, we sought to improve upon our published studies and more carefully characterise non-viral gene transfer to the kidney so that it can be applied by others in the field with expanded knowledge of the strengths and limitations of the technique.

Hydrodynamic tail vein injection is a highly effective non-viral method to transfect up to 40% of liver cells in rodents. Improved detection through the Clear, Unobstructed Brain/Body Imaging Cocktails and Computational analysis (CUBIC) protocol for tissue clearing found transfected cells in the heart, spleen, lung, stomach, intestines, and kidney, albeit many fewer than in the liver^7^. Since its concurrent discovery in 1999 by two groups^8^,^9^, the hydrodynamic tail vein injection method has gained popularity as one of the simplest ways to deliver transgenes to a mouse. Part of the success of the technique in the gene transfer field has been won by the inexpensive and simple materials required to perform it: 1) a buffered solution of endotoxin-free plasmid DNA 2) a restraining device 3) a method by which to heat the mice and 4) the correct syringe and needle gauge combination. After a short training with an experienced investigator, hydrodynamic tail vein injection can be quickly and safely done with low mortality. Hydrodynamic injection principles have been translated to other organs and large animals through the development of devices to introduce the DNA solution very quickly at an exact pressure^10^. Similar hydrodynamic injection methods have been successfully used to transfect rat and pig kidney^11^,^12^, however, the majority of the animal models of human disease have been generated in mice. Here we report on an inexpensive, fast, and reproducible method to achieve long-term gene expression in multiple cell types of the mouse kidney.

Short-term high levels of gene expression can be achieved by introducing plasmid DNA purified from bacteria by a variety of methods, both physical and chemical. However, both the silencing and degradation of plasmid DNA are important factors that hinder long-term gene expression. Transposons are mobile DNA elements that can be engineered such that the transgene is flanked by inverted repeat sequences and the transposase is expressed on a separate helper plasmid to catalyze integration of the transgene into the genome^13^. We hypothesised that the hyperactive *piggyBac* transposase would increase long-term gene expression since it has been shown to be highly active in many different cell types, including in mice *in vivo* following hydrodynamic tail vein injection^14^,^15^,^16^. The other important factor hindering long-term gene expression after almost all gene transfer techniques is an immune response to the protein product of the transgene. Luciferase immunogenicity has been well documented by our lab and others^17^,^18^,^19^. Treatment of mice with cyclophosphamide following hydrodynamic tail vein injection of *Sleeping Beauty* transposons attenuated the typically observed drop in transgene-encoded protein expression in the liver, so we followed a similar protocol here to prevent the loss of gene expression seen in the first few weeks following transgene introduction and effectively translated this approach to the kidney^20^.

## Results

### Hydrodynamic tail vein injection of luciferase constructs driven by kidney-specific promoters

Although hydrodynamic tail vein injection primarily transfects liver, there have been some reports of its use to generate phenotypic outcomes in the kidney. Mice given hydrodynamic tail vein injection to produce exogenous Wnt1 expression had more severe podocyte injury, but this report did not examine whether the source of the expressed Wnt1 was liver, kidney, or both^21^. Other reports of glomerular expression following hydrodynamic injection include plasmids expressing podocin-driven dynamin1 and Klf4 expressed from the cytomegalovirus (CMV) promoter^22^,^23^. Therefore, we hypothesised that the hydrodynamic tail vein injection method could be translated to the kidney by employing a kidney-specific promoter.

**Figure 1:**
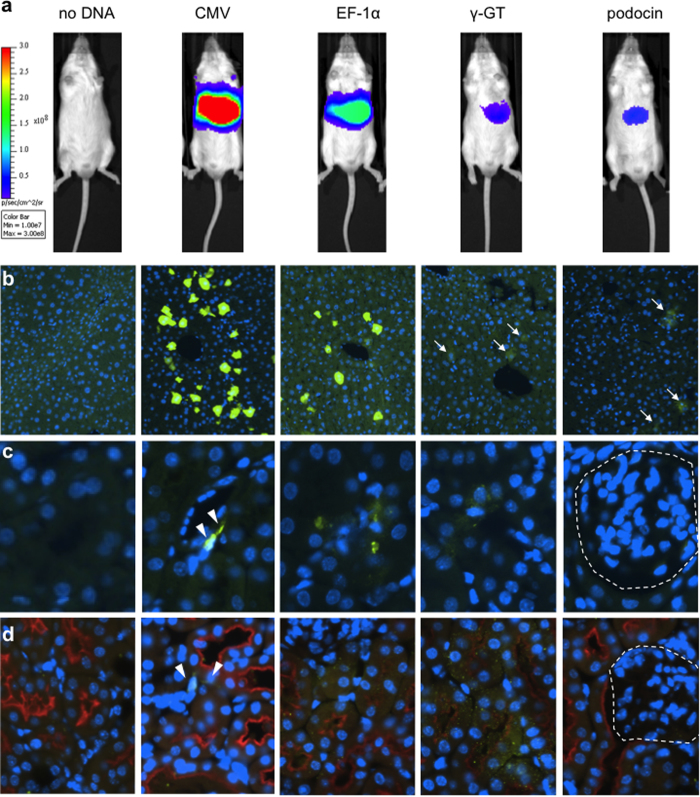
Hydrodynamic tail vein injection of plasmid DNA transgenes *in vivo* produces expression mainly in the liver with rare expression in the kidney. (**a**) Mice were injected with 20 μg of an enhanced firefly luciferase transposon driven by the promoter shown and imaged the following day to detect luciferase expression. The tissues were stained for luciferase (green) and nuclei by DAPI (blue). (**b**) Liver; faintly luciferase-positive cells are indicated by arrows. (**c**) Kidney; interstitial luciferase-positive cells are indicated by arrowheads and the glomerulus is marked by a dashed outline. (**d**) Co-staining in kidney with *SLC3A1*/rBAT to mark the proximal tubules (red). Representative of n = 3. Promoters used to express luciferase were: CMV (cytomegalovirus, constitutive viral), EF-1α (Elongation Factor-1 alpha, constitutive endogenous), γGT1 (Gamma-glutamyl transferase, tubule-specific), and podocin (podocyte-specific).

We tested four luciferase constructs all expressing enhanced firefly luciferase^24^ under the control of different promoters (Supplementary Figure S1). The promoters tested were the strong viral promoter CMV, the strong mammalian constitutive promoter elongation factor-1 alpha (EF-1α), the proximal tubule-specific promoter gamma-glutaryl transpeptidase (γGT1), and the podocyte-specific human podocin promoter (podocin). Both kidney-specific promoters are cell-type specific and have been fully validated in transgenic models^25^,^26^,^27^. One day after gene transfer, luciferase levels were highest for CMV, followed by EF-1α; γGT1 and podocin were lower and both appeared approximately equal. All four promoters expressed most strongly in the liver (Fig. 1a). We immunostained for luciferase (green) both in liver and kidney to determine the intensity of expression and number of transfected cells. We found the greatest number of positive cells in the livers of mice expressing the luciferase gene from the CMV promoter, followed by EF-1α, although cells of both types appeared similarly bright. In the livers of mice receiving the kidney-specific promoter plasmids, luciferase-expressing cells were visible but much fainter (Fig. 1b, arrows). In the kidney, we stained for luciferase alone (Fig. 1c) and co-stained for the amino acid transporter *SLC3A1*/rBAT to mark the luminal membrane of the proximal tubules (red, Fig. 1d). We observed positive cells from the CMV-luciferase hydrodynamic injection that appeared morphologically to be interstitial cells (Fig. 1c,d, arrowheads). Rare cells expressing luciferase in the kidney from the EF-1α and γGT1 promoters appeared to have diffuse staining that colocalized with rBAT-positive proximal tubules (Fig. 1d). We were unable to identify any cells in the glomerulus that were luciferase positive, including in mice administered a systemic injection of luciferase expressed from the podocin promoter (Fig. 1c,d, glomerulus indicated by the dashed line). From these data we conclude that hydrodynamic tail vein injection in healthy mice transfects a very low number of cells in the kidney and that introduction of a kidney-specific promoter was not sufficient to provide kidney-specific transgene expression.

### Development of the renal pelvis injection technique

Initially, we evaluated other forms of non-viral transfection of the kidney using the same pT-effLuc-Thy1.1 plasmid for all studies (Supplementary Figure S1). First, we injected lipophilic complexes composed of a commercially available polyethyleneimine (PEI) reagent and plasmid DNA by various routes of entry (Supplementary Figure S2a). Day 1 imaging of luciferase expression revealed localisation that appeared to be in the kidneys or lungs. Lung expression suggests leakage occurred from the renal injection into the vasculature, as tail vein injection of the complexes is known to give expression in the lungs^28^. One mouse given DNA-PEI and one mouse given uncomplexed DNA by renal hydrodynamic injection were sacrificed and subsequently imaged for luciferase expression. We found that the DNA-PEI complex-treated mouse had transfected cells in the kidney and adjacent areas of the peritoneum, while the mouse receiving DNA by renal hydrodynamic injection had higher expression that was localised to the kidney alone without any detectable expression in other tissues (Supplementary Figure S2b–d). The tissues transfected with the DNA-PEI complexes appeared to suffer from gross damage or disruption of normal fatty membranes inside the peritoneum. Mice receiving DNA-PEI complexes also had a high mortality immediately following surgery (~50%).

Another non-viral transfection technique that has been previously described is ultrasound-mediated microbubble technology^29^. DNA-containing microbubbles were introduced to the mouse via the tail vein and ultrasound was used to disrupt the microbubbles inside of the kidney^30^. This technique appeared safe as all mice were healthy following treatment. Luciferase expression in the mice was very high in some cases, but the locations of expression within the whole body and in the dissected organs were variable from mouse to mouse (Supplementary Figure S3). We were unable to confirm high levels of kidney expression in any of the six mice tested here (Supplementary Figure S3b). In conclusion, in contrast to the kidney-specific gene expression achieved by renal hydrodynamic injection (Supplementary Figure S2c), we found both direct kidney injections of DNA-PEI complexes and the ultrasound microbubble technique were not kidney-specific when a constitutive CMV promoter was used for gene expression (Supplementary Figures S2d, S3b). These other methods may be acceptable in some biological contexts; however, we sought to achieve a truly kidney-specific gene transfer method.

**Figure 2:**
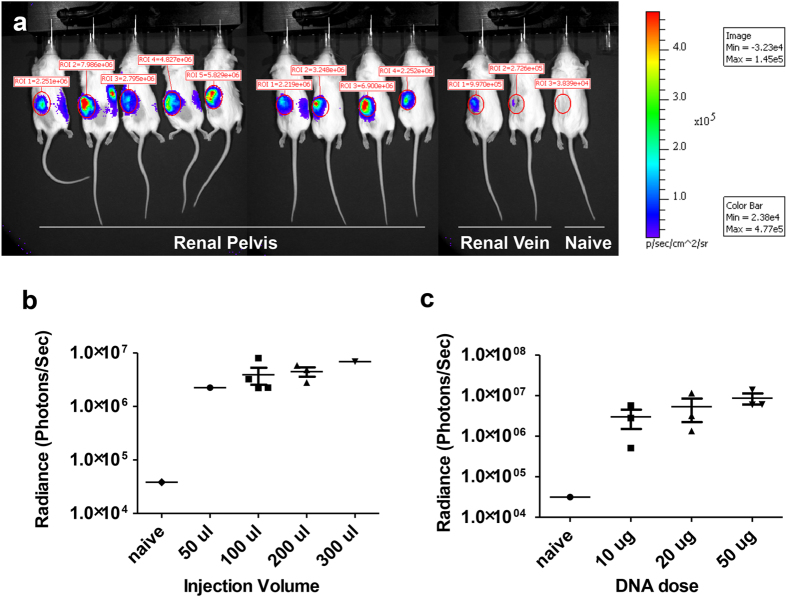
Kidney-specific gene transfer through different injection routes, volumes, and DNA doses. (**a**) Mice were injected in the renal pelvis or renal vein of the left kidney as indicated with 10 μg of pT-effLuc-Thy1.1 luciferase transposon and imaged the following day to detect luciferase expression. In (**b**) and (**c**), the photons/second emitted from the left kidney were quantified for various injection conditions. (**b**) Mice were injected in the renal pelvis with varying volumes of injection fluid while the transposon amount was held constant at 10 μg. (**c**) Mice were given varying doses of DNA in 100 μl volume (n = 3).

We optimised hydrodynamic injection to the kidney by defining the route of entry, injection volume, and DNA dose. We found three major points favoring direct injection into the renal pelvis compared to renal vein injection: (1) in renal vein injections, the needle should be as small as possible to minimise vein damage, but the small needle size works in opposition to the goal of a very fast injection; (2) injections into the renal pelvis had low mortality, while up to 50% of the mice subjected to renal vein injection died, presumably from bleeding from the injection site; and 3) luciferase expression was approximately one order of magnitude higher after renal pelvis injection than after renal vein injection (Fig. 2a). Thus, the renal pelvis proved a superior route of entry compared to the renal vein.

We evaluated the effect of different volumes and DNA doses on luciferase expression in mice injected into the renal pelvis. In Fig. 2a, mice injected in the renal pelvis were all given 10 μg of a CMV-expressed luciferase transposon diluted into different volumes of injection buffer. As the volume of the injection increased, the luciferase expression displayed an upward trend, but this difference was not significant (Fig. 2b). Transgene expression was equivalent with 100 μl compared to 200 μl. During hydrodynamic tail vein injection the liver rapidly expands, so we expected the kidney to swell in response to hydrodynamic kidney injections as well^31^. Regardless of volume, after some of the injections fluid leaked into the capsule and haematomas were observed. With an injection volume of 300 μl, we sometimes observed bursting of the kidney. Therefore, we determined that 100 μl was the optimal injection volume to minimise damage while maintaining a high level of gene expression. We then tested the effect of DNA dose on luciferase expression using a constant volume of 100 μl. We found that while increasing the DNA dose from 10 μg to 50 μg resulted in an upward trend, the impact on gene expression was not statistically significant (Fig. 2c).

### Localisation of injection and gene delivery to the kidney

**Figure 3:**
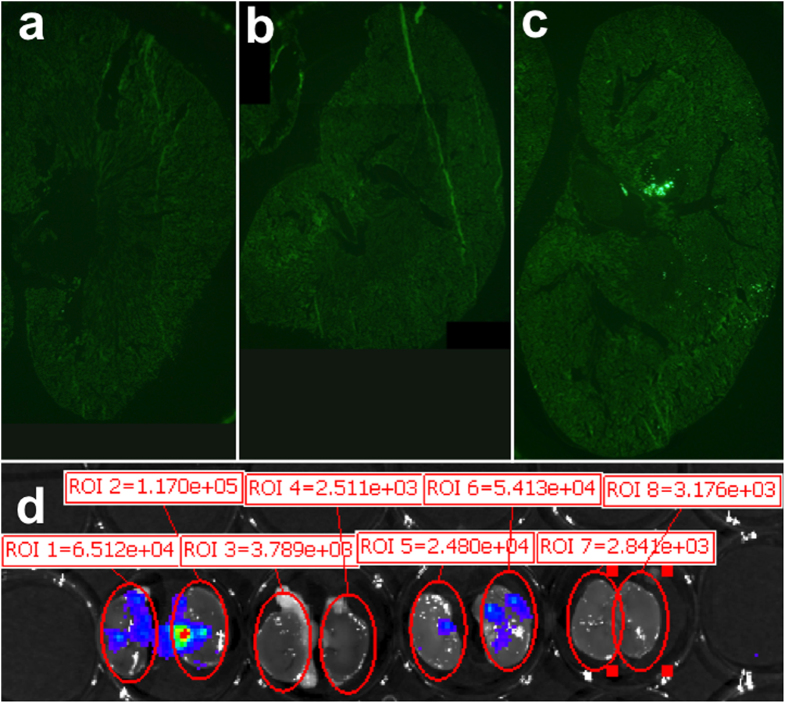
Renal pelvis injection reaches both medulla and cortex result in punctate areas of gene expression. (**a**) Uninjected kidney. (**b**) QR buffer-injected kidney. (**c**) Injection of latex fluorescent microspheres in a 100 μl volume of QR buffer. Two fields of view were assembled to display the entire kidney section. (**d**) IVIS image showing gross areas of punctate gene expression in bisected kidneys six days following DNA injection of 10 μg of luciferase transposon into the renal pelvis. Shown are both bisected kidneys from two animals: the left kidney was injected with DNA while the right kidney was uninjected. Each kidney half is arranged with the renal pelvis pointing inward toward the other half and the cortex on the exterior.

In order to determine where the renal pelvis injection distributed within the kidney, we injected mice with fluorescent microspheres to label the injection path (Fig. 3a–c)^32^. Evaluation of an entire cross-section of the kidney showed an uneven distribution of the microspheres. A large number of microspheres were detected in the medullary area, while ray-like sections of distribution were observed extending throughout the cortex to the capsule, in which an appreciable number of beads could be found. During injection, we observed that the kidney changed color from red to white in one or more areas constituting about one-third of the kidney, while other areas maintained a normal appearance during injection.

To grossly determine the location of luciferase expression in mice receiving a CMV-driven luciferase plasmid, mice were dissected following imaging and their kidneys were bisected and imaged on the *In Vivo* Imaging System (IVIS) machine (Fig. 3d). Six days following renal hydrodynamic injection, punctate areas of gene expression could be observed in both the medulla and cortex of kidneys from both mice. Similar to the distribution of beads shown in Fig. 3c, there are many blank areas in which no gene expression could be found. From these data, we concluded that the gene expression induced by renal pelvis injection occurred in patches of cells that expressed luciferase at high levels.

**Figure 4:**
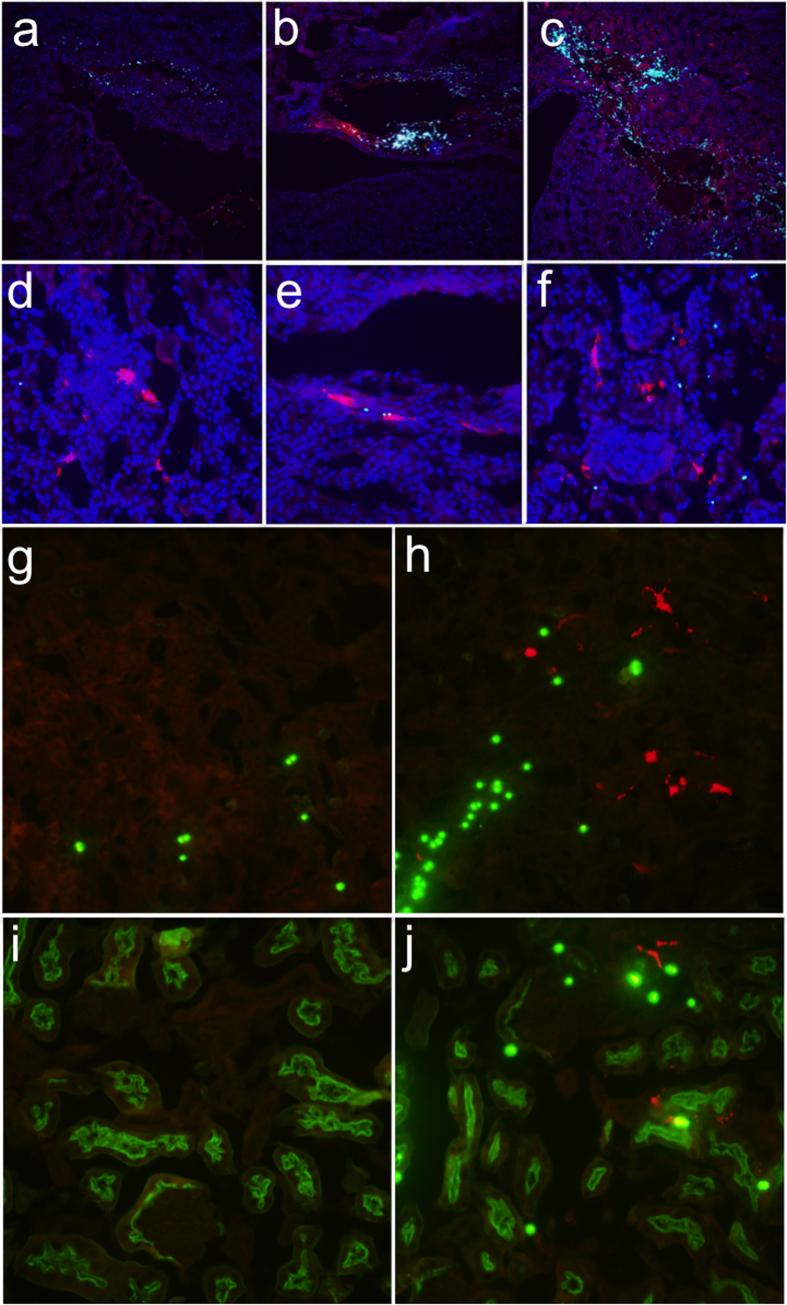
Renal pelvis injection results in expression in multiple kidney cell types. Anesthetised mice were injected in the renal pelvis with a 100 μl solution containing latex fluorescent microspheres without (**a**) or with (**b,c**) 20 μg pEF-1α-TdTomato (n = 3). High concentrations of beads (small cyan circles) were found within damaged areas (**a–c**). In the presence of DNA, the areas of damage were colocalised with TdTomato (red) (**b,c**). TdTomato-positive cells in the glomerulus (**d**), tubules (**d–f**), and collecting duct (**e**), colocalised with beads. In (**a–f**) nuclei were counterstained with DAPI (blue). (**g–j**) Sections costained for TdTomato (red) and fluorescein lotus lectin (green) to mark the proximal tubules. Microspheres appear as bright green circles. (**g**) Medulla of mouse receiving beads alone. (**h**) Medulla of mouse receiving beads and pEF-1α-TdTomato. (**i**) Cortex of mouse receiving beads alone. (**j**) Cortex of mouse receiving beads and pEF-1α-TdTomato.

To further localise cell types transfected by renal pelvis injection, we injected mice with 20 μg of pEF-1α-TdTomato plasmid since the expression of luciferase in possible tubules from the EF-1α promoter was more desirable than the expression from interstitial cells observed for the CMV promoter (Fig. 1c,d). TdTomato is a derivative of the red fluorescent protein (RFP) so we used an anti-RFP antibody to amplify the signal. In Fig. 4a–f, transgene expression is shown in red, nuclei are labelled with DAPI in blue, and fluorescent microbeads were detected on all three channels and therefore have a light cyan appearance in the merged images. In mice injected with beads alone, the beads were co-localised with areas of damage but no bright red cells were found (Fig. 4a). In contrast, in mice given beads and the pEF-1α-TdTomato plasmid by renal pelvis injection, beads and transfected cells are found in the same areas, generally with a “halo” effect in which the cells surround the areas littered with beads. Not shown are many areas in the injected mouse kidney in which the histology was completely normal, and no beads or transfected cells could be found. The expression of TdTomato was heterogeneous, as in Fig. 3, and co-localised heavily with the fluorescent microspheres. Quantification of the transfection efficiency was impeded by the variable gene expression in different areas of the kidney and the high background in the kidney due to autofluorescence of metabolites such that only the highest-expressing cells could be confidently counted as positive, although the actual numbers may have been higher. Overall the transfection efficiency was substantially lower (<1%) than that achieved in the liver by hydrodynamic tail vein injection (<40%).

Higher magnification images in Fig. 4d–f show highly expressing TdTomato^(+)^ cells within the glomerulus (Fig. 4d; very rare), and medullary and cortical tubules (Fig. 4e–f, respectively). In order to determine the types of tubules that were transfected, we co-stained with fluorescein lotus lectin (green) that only stains proximal tubules. In both the medulla (Fig. 4g,h) and the cortex (Fig. 4i,j), cells that were positive for the TdTomato transgene (red) colocalised with fluorescent microbeads (green spheres) and were only found in mice injected with the pEF-1α-TdTomato plasmid (Fig. 4h,j). Transfected cells are localised to lotus lectin-negative areas of the kidney, indicating transfection of non-proximal tubular cells (Fig. 4h). There are also transfected cells within both lotus lectin-positive and lotus lectin-negative tubules, suggesting both the proximal and distal tubule sections were transfected via renal pelvis injection (Fig. 4j). We conclude that the renal pelvis injection technique is not cell type specific, a finding that is indicative of the mechanical nature of the gene transfer. Rather than requiring a receptor or other biological path for cellular entry, the renal hydrodynamic injection transfected kidney cells of any type that were within the path and subjected to sufficient force to induce entry of the plasmid DNA. However, the use of kidney cell-type specific promoters could potentially improve cell-type specificity of transgene expression.

### Renal pelvis injection induced temporary kidney damage

**Figure 5:**
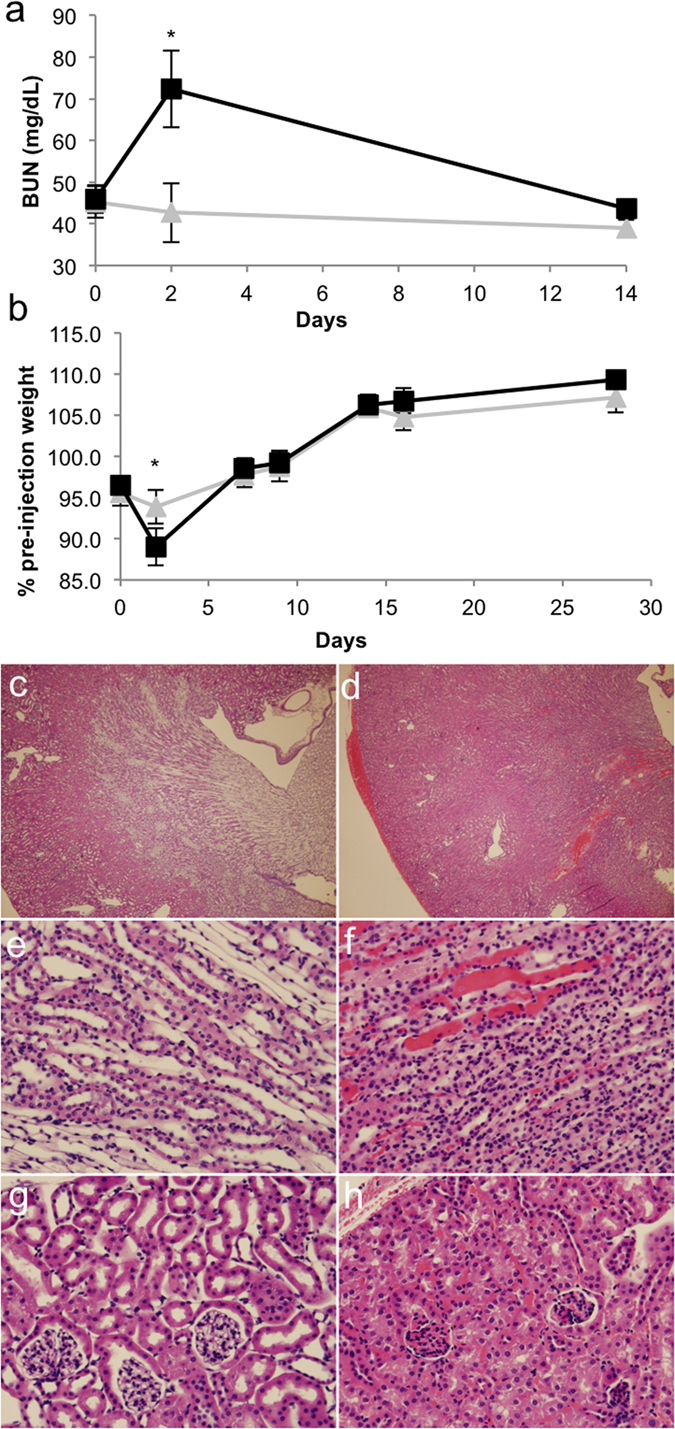
Hydrodynamic renal pelvis injection causes transient kidney injury. Mice in (**a,b**) were given a unilateral nephrectomy to remove the right kidney. One week later, mice were subjected to either gene transfer (black squares; n = 5) or sham surgery (grey triangles; n = 3). Sham mice were anesthetised and an incision was made but the kidney was not injected. (**a**) Serum was collected prior to gene transfer (Day 0), 2 days post-gene transfer, or 14 days post-gene transfer and blood urea nitrogen (BUN) was measured. The asterisk indicates statistical significance at Day 2 (*p* = 0.02, one-tailed Student’s *t*-test assuming unequal variance). (**b**) The body weight was measured over time and compared to the pre-nephrectomy original body weight of the mice. The asterisk indicates statistical significance at Day 2 (*p* = 0.04, one-tailed Student’s *t*-test assuming unequal variance). H&E staining of kidneys taken from either (**c**,**e**,**g**) naïve littermate mouse or (**d**,**f**,**h)** mouse receiving a renal pelvis injection of 10 μg pT-EeL (EF-1α-luciferase) transposon one day prior and harvested following perfusion to reduce vascular erythrocytes present in the sections. (**c,d**) Overview showing the appearance of a subcapsular hematoma and increase in pockets of trapped erythrocytes present in the injected kidney. (**e**,**f**) Medulla and (**g**,**h**) cortex. Some tubular necrosis is present in (**h**) as indicated by white vacuoles.

It is well established that elevated levels of the liver enzymes alanine aminotransferase (ALT) and aspartate aminotransferase (AST) are found in the blood in the days following hydrodynamic tail vein injection^33^. The elevation of liver enzymes resolves within a week, a finding confirmed by histological examination of the tissues^34^. In preclinical studies, an increase in AST in pigs indicated that the injection was given at an appropriate speed and pressure to produce robust transgene expression, although less increase in ALT was seen^35^. Taken together with our own gross observations of kidney damage incurred during renal pelvis injection, we hypothesised that renal pelvis injection damaged the kidney; however, similar to the liver, the damage likely resolved quickly. Mice given renal pelvis injections of inert marker transgenes such as luciferase were usually quite healthy, presumably because the uninjected kidney could compensate for any decrease in function in the treated kidney. Therefore, to measure the extent and persistence of kidney damage induced by renal pelvis injection, we performed unilateral nephrectomies on mice and injected the remaining kidney one week later. Uninephrectomised sham-treated mice received anaesthesia and the surgical incision but not the renal pelvis injection. Blood urea nitrogen (BUN) was significantly elevated at the Day 2 timepoint in mice receiving the injection (*p* = 0.02), but not at Day 0 (*p* = 0.5; after unilateral nephrectomy but before injection) or at the later Day 14 timepoint (*p* = 0.09; Fig. 5a). Similarly, there was more weight loss in mice receiving the injection at Day 2 following surgery (*p* = 0.04), but these animals quickly gained body weight and appeared identical to the sham group by one week after renal pelvis injection (Fig. 5b).

To examine the histology of the injected kidneys, we injected littermate mice with 10 μg of a transposon expressing luciferase from the EF-1α promoter. Mice were sacrificed the following day and their kidneys harvested following cardiac perfusion. Paraffin-embedded kidney sections were stained with haematoxylin and eosin (H&E) and evaluated for histological changes. Mice given the injection had haematomas under the kidney capsule and within the kidney parenchyma (Fig. 5d) that were not present in naïve kidneys (Fig. 5c). Within the medulla and cortex of the injected kidney, erythryocytes were present in the interstitium (Fig. 5e–h). White vacuoles were found within the tubules of the injected kidneys indicative of tubular injury (Fig. 5g,h). In summary, mice receiving a renal pelvis injection experienced kidney damage, but BUN analysis indicates that this damage resolved within 14 days.

### Effect of transposon integration and promoter choice on long-term transgene expression

**Figure 6:**
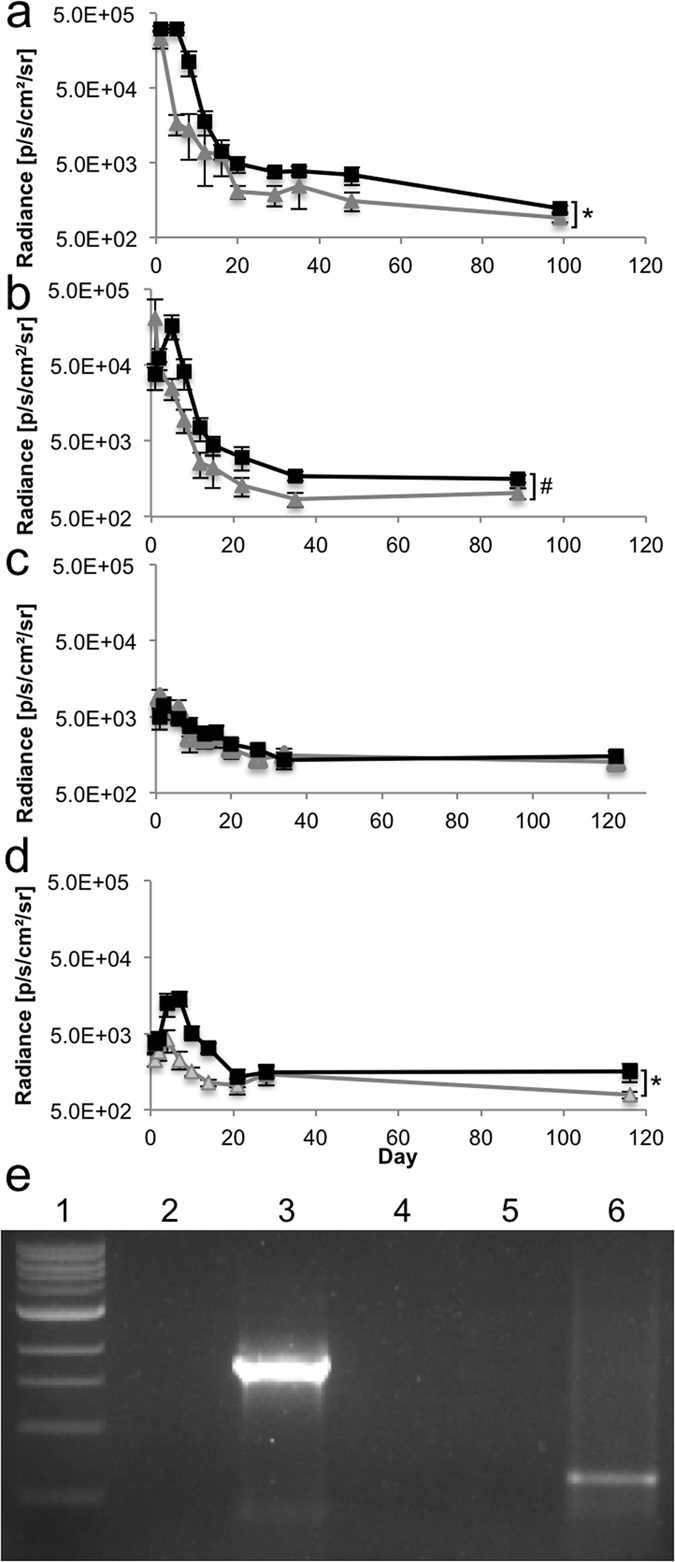
*piggyBac* transposition in the adult mouse kidney. Mice received either 50 μg of the *piggyBac* enhanced firefly luciferase transposon alone (grey triangles) or transposon plus 10 μg of the hyperactive *piggyBac* transposase (black squares) by renal pelvis injection. The plasmids expressed both enhanced firefly luciferase and hyperactive *piggyBac* transposase from the following promoters: (**a**) CMV (transposon alone, n = 4; with transposase, n = 8), (**b**) EF-1α (transposon alone, n = 5; with transposase, n = 4), (**c**) γGT1 (transposon alone, n = 4; with transposase, n = 5), and (**d**) podocin (transposon alone, n = 4; with transposase, n = 5). Either an asterisk (*p < 0.05 by Mann-Whitney U test comparing the area under the curve for each mouse as calculated by the trapezoidal approximation rule) or a pound sign (^#^*p* < 0.05 by Student’s *t*-test at the last timepoint) indicate statistical significance. (**e**) Excision assay to detect the molecular junction remaining in the transposon plasmid after the transposon has been removed by active *piggyBac* transposase. An agarose gel is shown in which lane 1 is the 100 bp ladder and the remaining lanes were each loaded with the same amount of PCR product from a reaction in which the template DNA was either: (2) water, (3) positive control, 4) liver, 5) uninjected kidney, or 6) injected kidney removed from a mouse injected 24 hours prior as in the transposase group in (**a**). The positive control band is larger than the band in the injected kidney lane because the control was made by digesting the transposon plasmid to remove the majority of transposon sequences and self-ligating the backbone plasmid. This procedure created a DNA template for the PCR reaction that gave a slightly larger PCR product than that produced by direct *piggyBac* transposon excision.

To optimise transgene expression in the kidney at later timepoints, we performed renal pelvis injection of the various helper and donor plasmid sets and measured kidney-specific expression over time by live animal imaging (Supplementary Figure 1 and Fig. 6). For each pair, both the hyperactive *piggyBac* transposase and the enhanced firefly luciferase carried by the transposon were expressed from the same promoter. We used two separate statistical tests to evaluate if the groups were different. By calculating the area under the curve for each mouse and comparing the groups by Mann-Whitney test, we could assess overall changes in expression. We also compared the last timepoint by Student’s *t*-test to detect differences in long-term gene expression. For the CMV promoter, luciferase levels were higher with the transposase for all timepoints by Mann-Whitney comparison of the areas under the curve (*p* = 0.004) but the last timepoint *t*-test *p*-value was not significant (Fig. 6a). For the endogenous promoter EF-1α, the *piggyBac* transposase group started off much lower than the group given transposon alone, then increased greatly shortly after gene expression, followed by a steady decline that mirrored the group not given transposase (Fig. 6b). We were mostly interested in changes in long-term gene expression. Comparison of the area under the curve for mice in each group from Day 5 onward was significant (*p* = 0.02) (Fig. 6b). EF-1α was the only promoter for which a statistically significant difference at the last timepoint was found with a 1.5-fold increase at Day 89. The proximal tubule specific γGT1 promoter is notable as the only promoter for which there was never an increase in the luciferase level in the group including the hyperactive *piggyBac* transposase (Fig. 6c). The podocin groups were interesting in that they both started off low, increased to a peak at about one week post-gene transfer, then steadily declined again, producing a difference in the groups that was significant overall by Mann-Whitney test for the areas under the curves (*p* = 0.02) but at the last timepoint was not statistically significant (Fig. 6d). Of the promoters we tested, the EF-1α promoter seemed best for long-term expression of both the delivered transgenes and the *piggyBac* transposase in the kidney.

To molecularly confirm that the *piggyBac* transposase was functional, we tested if the transposon was excised in the injected kidney by excision PCR assay. To perform this assay, we injected mice with the luciferase transposon and transposase plasmids then isolated organs the following day. DNA was purified from the liver, uninjected kidney, and injected kidney for the excision PCR template. For the excision PCR, we amplified a short region of DNA spanning the portion from which the transposon was excised. Since a short extension time was used, a band was seen only if the PCR product was small enough to be amplified (Fig. 6e, Lane 6, injected kidney) but no band would be present if the transposase did not excise the transposon or there were no transposon sequences present (Fig. 6e, Lane 5, uninjected kidney). The band in Lane 6 was purified and sequenced to further verify transposition. From this assay we concluded that the *piggyBac* transposase was able to cut the transposon from the donor plasmid in the kidneys of live mice *in vivo*.

### Expression of erythropoietin from the kidney after renal pelvis injection increased the haematocrit

**Figure 7:**
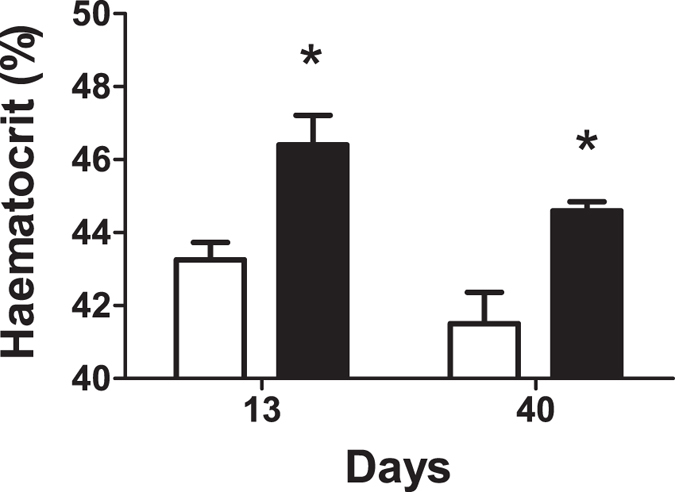
Renal pelvis injection of a transposon expressing erythropoietin increased the haematocrit. Mice received a renal pelvis injection of 10 μg of hyperactive *piggyBac* plasmid with 50 μg of either enhanced firefly luciferase (white bars, n = 4) or erythropoietin (black bars, n = 5) transposon, each expressed from the EF-1α promoter. The mice were bled at the number of days following the injection indicated on the graph to determine the haematocrit. *p < 0.05 by one-tailed Student’s *t*-test with unequal variance.

To test the ability of renal pelvis injection to deliver a transgene having a biological effect, we created a *piggyBac* transposon expressing the hormone erythropoietin (Epo) to drive red blood cell production. We performed renal pelvis injection in mice with either this plasmid or the *piggyBac* transposon expressing the enhanced firefly luciferase gene from the same EF-1α promoter and hyperactive *piggyBac* transposase helper plasmid. We expected expression to increase the number of red blood cells as measured by the haematocrit and found that at 13 days post-gene transfer, the mice given the Epo transposons had a significantly increased haematocrit as compared to those given luciferase (*p* = 0.007 by one-tailed Student’s *t*-test with unequal variance; Fig. 7). By 40 days after gene transfer the difference between sham luciferase and Epo-treated was still significant (*p* = 0.02 by one-tailed Student’s *t*-test with unequal variance). As the only difference in treatment between the two groups was whether the Epo or luciferase gene was expressed from the EF-1α promoter on the transposon we conclude that the haematocrit elevation resulted directly from the expression of the hormone Epo following renal pelvis hydrodynamic injection.

### Cyclophosphamide prevented a decrease in long-term gene expression over time

**Figure 8:**
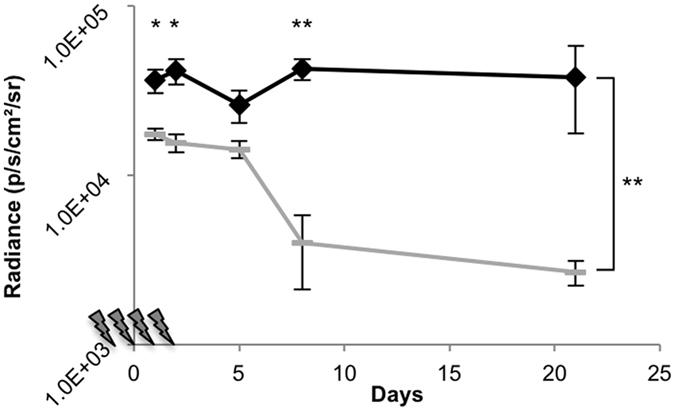
Cyclophosphamide treatment for immunosuppression resulted in increased luciferase expression. Mice were given either saline (grey dashes, n = 3) or four cyclosphosphamide treatments (black diamonds, n = 4) during the week of the renal pelvis injection. Lightning bolts on the x-axis represent each cyclophosphamide injection. Each mouse received a renal pelvis injection of 10 μg of hyperactive *piggyBac* plasmid with 50 μg of enhanced firefly luciferase transposon, each expressed from the EF-1α promoter. Radiance was determined by live animal imaging on the IVIS machine at the days indicated following the renal pelvis injection. Bracket indicates comparison of the areas under the curve. *p < 0.05, **p < 0.005 by Student’s one-tailed *t*-test assuming unequal variances.

Even with the EF-1α promoter for gene expression and hyperactive *piggyBac* transposase for integration of the luciferase transgene, the absolute levels of gene expression dropped by two orders of magnitude within a few weeks of injection (Fig. 6b). This result is similar to that observed in the liver^9^. Others have shown that treatment with cyclophosphamide prolonged luciferase expression in the liver when delivered on *Sleeping Beauty* transposons via hydrodynamic injection^20^, so we tested if cyclophosphamide could also prolong gene expression in the kidney. Cyclophosphamide is a potent immunosuppressive reagent that kills T regulatory CD4 + CD25 + T cells when administered at high doses. To test the hypothesis that T cell-induced killing of transgene-expressing kidney cells causes the drop in gene expression observed over the first few weeks after administration, we treated mice with four doses of cyclophosphamide around the time of renal pelvis injection (24 hours prior, immediately following surgery, 24 and 48 hours after injection) according to the previously published protocol^20^. We observed sustained maintenance of gene expression during the first few weeks following renal pelvis injection in mice receiving cyclophosphamide as compared to saline-treated controls (Fig. 8). This effect was statistically significant (*p* < 0.05) by one-tailed t-tests assuming unequal variance for three out of five timepoints (Day 1, *p* = 0.02; Day 2, *p* = 0.02; Day 5, *p* = 0.06; Day 8, *p* = 0.003; Day 21, *p* = 0.09) and by comparison of the areas under the curve (*p* = 0.005).

## Discussion

We found that hydrodynamic tail vein injection, a predominantly liver-targeting gene delivery method, produced rare luciferase-positive cells in the kidney (Fig. 1). Others have used hydrodynamic tail vein injection in combination with a kidney-specific promoter in their studies^22^,^23^. Our results reveal that while this may produce a low level of kidney gene expression, the majority of the transgene expression still occurs in the liver even when using kidney-specific promoters. We also demonstrated that our technique achieves superior kidney-specific transgene expression when compared to the ultrasound-microbubble technique or the use of PEI.

The renal pelvis injection method produced patches of gene expression and was not cell-type specific. The epigenetic programming that occurs during normal development is missing when gene transfer is performed in the adult mouse, so the factors needed to silence the promoter in mismatched cell types may not be present in the adult at the levels required to achieve a truly cell type-specific promoter. To circumvent this issue, perhaps designer promoters could be found by screening that would achieve greater specificity following gene transfer in the adult mouse, as has been done in the liver with computationally enhanced promoters^16^. It remains to be determined if the use of more specific, computer-designed promoters could enable expression in only the desired kidney cell type by renal pelvis injection.

Although transgene expression dropped off greatly over time, both in the absence and presence of *piggyBac* transposase (Fig. 6), we were able to increase haematocrit significantly when the hormone Epo was delivered to the kidney via renal pelvis injection as compared to luciferase (*p* = 0.007; Fig. 7). At 40 days post-injection, the haematocrit of the Epo-treated animals was still elevated relative to luciferase-treated sham control mice (*p* = 0.02), although it was lower than at 13 days post-treatment. It is possible that gene silencing and/or cell death in the population of Epo-transfected cells resulted in this, as the immune response to mouse Epo in mice should be low as it is not a foreign protein. Many desired transgene-expressed proteins are foreign to the immune system, such as firefly luciferase. To prolong expression of foreign genes and prevent the steep drop-off in gene expression that consistently occurred over the first several weeks, we successfully suppressed the immune system with cyclophosphamide (Fig. 8). This not only points to the most dominant mechanism of the drop in transgene-expressed protein products but also indicates a future direction for increasing long-term gene expression: refinement of immune system modulation. In the future we plan to carefully test different methods and protocols to circumvent the regulatory T cell population and allow cells expressing the transgene-encoded protein product to persist long-term.

Finally, we offer some comments on the applicability of the renal pelvis injection technique. The purpose of this study was to create a new method by which to test the feasibility of kidney-specific delivery in adult mice. While efficiency was low, we anticipate that renal pelvis injection could be used to transfect difficult cell types and remove them from the animal for propagation or study, to create rare cells containing oncogenes or reprogramming factors, or to study if mutant genes are able to functionally compensate in knockout mouse models. Renal pelvis injection could also be used for studies of the expression of secreted factors that must be post-translationally modified by the kidney. The elevation of blood urea nitrogen and drop in weight that we observed in the mice given a unilateral nephrectomy prior to surgery, suggests that kidney damage occurred. Damage similarly occurs when using hydrodynamic injection technology to transfect the liver in mice. Our method should not be seen as a substitute for making a transgenic mouse that will express the gene of interest in the correct cell type throughout the organ. Nonetheless, kidney-specific transposon-mediated gene transfer will enable exploration of new research questions in adult mice and may be used for proof-of-concept gene transfer studies.

## Materials and Methods

### Mice

Male FVB mice (Strain 207; Charles River, Cleveland, OH), were used for all studies except in Fig. 5c–h where 129SV and C57Bl/6 mixed background, littermate mice bred in-house were used. Mice in all experiments were 2–4 months old at the time of injection. All animal procedures were conducted in accordance with the relevant guidelines and regulations and approved by the Institutional Animal Care and Use Committees of Baylor College of Medicine, Vanderbilt University Medical Center, and the Department of Veterans Affairs.

### Plasmids

The transposon vector expressing enhanced firefly luciferase^24^ from the CMV promoter has been described previously as pT-effLuc-Thy1.1^36^. The previously described pCMV-HA-m7pB vector^15^ drives hyperactive *piggyBac* transposase from the CMV promoter. An intermediate plasmid termed pTtight-effLuc was made by blunting with Klenow such that the NcoI-HF/EcoRI effLuc fragment from pT-effLuc-Thy1.1 replaced the Luc in pTtightLuc by digest with XmaI and NheI^28^. To create pT-effLuc, PCR of the effLuc gene was performed with primers RemoveTight-F1: 5′GTAACTCGAGCTAGCGGCGCGCCACGCGTCACGTGCGCCTGGAGAATTCGAG and RemoveTight-R1: 5′GACTTTCCACACCCTAACTG to add restriction sites. Both this PCR product and pTtight-effLuc were XhoI digested and the ligation product was pT-effLuc. The EF-1α promoter was obtained by liberating a fragment from Addgene plasmid 11154, pEF-EGFP^37^, with HincII and cloning into PmlI digested pT-effLuc to make pT-EF-1α-effLuc. This was then digested with AbsI to remove a superfluous neomycin resistance cassette to create pTEeL. To make pTGeL, the γGT242.2 plasmid containing the rat *γGT1* promoter^26^ was cut with EcoRI and XbaI and blunted to give the γGT1 promoter fragment which was cloned into pTEeL digested with MluI and NcoI-HF and blunted. pTEeL and pHP2.5Luc containing a 2.5-kb fragment of the human *NPHS2* promoter^38^ were digested with MluI and NcoI-HF and ligated to make pTPeL. To make pEF-1α-HA-m7pB, pγGT1-HA-m7pB, and pHP2.5-HA-m7pB, AscI and NcoI were used to digest pTEeL, pTGeL, and pTPeL and the Klenow-blunted promoter fragments were cloned into a blunted EcoRI and BbsI digested pCMV-HA-m7pB vector. Cloning enzymes (New England Biolabs, Ipswich, MA) and pEF-1α-TdTomato (Clontech, Mountain View, CA) were purchased.

### Hydrodynamic tail vein injections

Hydrodynamic tail vein injections were carried out as previously described^28^. Briefly, 20 μg of luciferase-expressing plasmid was diluted in 1.8 mL of QR buffer (Mirus Bio, Madison, WI) and quickly injected into the tail vein via a 27 gauge needle.

### Renal pelvis injections

Mice were anesthetised with ketamine (120 mg/kg) and xylazine (20 mg/kg) administered by intraperitoneal injection, placed on a water-circulated heat pad (Kent Scientific, Torrington, CT), and shaved. For renal vein injections, the vein was visualised through an abdominal incision and injected with a 15 mm 33 gauge micro cannulation needle (Fine Science Tools, Foster City, CA) sealed to a syringe with Parafilm M (Bemis Co., Oshkosh, WI). For renal pelvis injections, initial studies (Fig. 2) involved both abdominal and flank incisions injected from either side of the renal pelvis, but flank incisions were always used in the following studies. For these, a dorsal incision was made and the kidney pushed out of the peritoneum without directly manipulating it. The renal hilum was gently separated from surrounding fat only if necessary for visualization of the renal pelvis, located as the small white area on the lower medial side of the kidney, visualised by gently pressing the kidney down with closed forceps. Injections were performed with a 29 G 0.5 mL insulin syringe without a safety shield (#324703, BD, Franklin Lakes, NJ) in 1–3 seconds. The needle was kept in place for approximately five additional seconds and then removed from the renal pelvis. The kidneys were returned to the abdomen and mice were sutured with 5–0 coated vicryl violet braided 17mm 1/2c taper sutures (Ethicon, Somerville, NJ) for the muscle layer and 5–0 prolene blue monofilament 17 mm 1/2c taper sutures (Ethicon) for the skin layer. As mice returned to consciousness they were treated with a subcutaneous injection of buprenorphine followed by three additional doses to provide pain control during the first 48 hours of the post-operative period.

### Live animal imaging

Mice were imaged on the Xenogen IVIS 200 (Perkin Elmer, Waltham, MA) approximately five minutes after injection with 100 μg of luciferin substrate (Perkin Elmer, Waltham, MA) in phosphate buffered saline without calcium or magnesium (PBS; Corning, Manassas, VA) as previously described^19^.

### Histology and immunostaining

For paraffin-embedded sections, cardiac perfusion was performed with approximately 30 mL of PBS followed by freshly prepared 4% paraformaldehyde in PBS one day post-injection. For luciferase and *SLC3A1*/rBAT staining, livers and bisected kidneys were harvested on ice and fixed overnight in 4% paraformaldehyde at 4 °C. The tissues were paraffin-embedded and sectioned by the Pathology and Histology Core at Baylor College of Medicine. Fluorescent staining of paraffin-embedded sections was performed as previously described with the following antibodies and dilutions, for Fig. 1c: (1) goat anti-luciferase (G745A, Promega, Madison, WI) 1:50; (2) alexafluor donkey anti-goat 488 (Life Technologies, Carlsbad, CA) 1:500; and for Fig. 1d: (3) rabbit anti-*SLC3A1* (16343-1-AP, Proteintech, Chicago, IL) 1:200 (4) donkey anti-rabbit 594 (Life Technologies) 1:500^19^. Primary antibodies were incubated sequentially overnight with a 15 min 4% paraformaldehyde fixation step performed between the first secondary antibody and the second primary antibody, followed by four washes. For H&E staining, kidneys were removed, bisected, and placed into 4% paraformaldehyde at 4 °C overnight with rocking. The tissues were paraffin-embedded, sectioned, and stained by the Translational Pathology Shared Resource core at Vanderbilt.

For frozen sections, mice were renal pelvis injected with Fluoresbrite Plain YG 2.0 Micron Microspheres (Polysciences Inc, Warrington, PA)^32^ with or without pEF-1α-TdTomato plasmid DNA. After 48 hours, mice were sacrificed and kidneys removed and bisected. They were kept in 4% paraformaldehyde in PBS at 4 °C for 6 hours followed by storage in sterile 30% sucrose at 4 °C. Kidneys were embedded in OCT compound, cut, and stained. Blocking and staining steps were performed in PBS buffer containing 10% donkey serum (Sigma) and 0.1% Triton X-100. For staining of transfected cells and nuclei, slides were incubated in serum buffer one hour, washed thrice in PBS, incubated with rabbit polyclonal anti-RFP (1:200 dilution, #PM005; MBL International Corporation, Woburn, MA), washed thrice in PBS, incubated with Alexafluor donkey anti-rabbit 594 (1:500 dilution; Life Technologies) and mounted in ProLong Gold with DAPI (Life Technologies). For staining of proximal tubules and transfected cells, blocked slides were incubated 20 minutes with fluorescein lotus lectin (1:200 dilution; Vector Laboratories Inc, Burlingame, CA), stained for TdTomato with the RFP antibody set as before, and mounted in ProLong Gold without DAPI (Life Technologies). Images were acquired on an Olympus BX51 microscope.

### Excision assay

Mouse organs were harvested one day following renal pelvis injection. They were minced with a straight razor on a sterile petri dish and plasmid DNA was extracted from 25 mg of tissue resuspended in 600 μl of PBS by miniprep with an extended five-minute neutralization step (Zymo, Seattle, WA). Excision PCR was performed with Taq ProRed (Denville Scientific, South Plainfield, NJ) in a 50 μl reaction with primers pBZeoexcF2 (ACCTTAGAGGCTATTTAAGTTG) and pBZeoexcR1 (GCTGCTCTCCAATCTCTGTC) containing 5 μl of template DNA extracted from liver or kidney, or 1 μl of positive control at a 60 °C annealling temperature. The positive control consisted of 5 μg of pTZeoeffLucThy1.1 that was digested with NsiI to remove the majority of the transposon sequences and ligated with T4 ligase in a 1 ml reaction to promote recircularization, then heat-killed at 70 °C. PCR products were confirmed by Sanger sequencing (Lone Star Labs, Houston, TX).

### Unilateral nephrectomy

Right kidneys were excised from the animal as for the renal pelvis injections. Tissues were carefully dissected away from the poles of the kidneys to preserve the adrenal gland and a 4–0 silk suture (Ethicon) was used to ligate the vessels with two surgical knots. The kidney was removed leaving a renal vascular stump which was monitored for bleeding prior to placement back into the body of the animal. Mice were given 0.5 mL normal saline immediately following surgery and buprenex for pain for 48 hours post-surgery by subcutaneous injections.

### Blood urea nitrogen

Blood obtained by tail clip was clotted for 30 minutes at room temperature in an eppendorf tube and centrifuged at 4500 rpm for 10 minutes to obtain serum samples. After collection was complete the serum samples were submitted to the Vanderbilt University Medical Center Comparative Pathology Laboratory for analysis of blood urea nitrogen.

### Cyclophosphamide treatment

On the day of the first injection, 25 mL of 0.9% normal saline (APP Pharmaceuticals, Schaumburg, IL) was added to a 500-mg bottle of cyclophosphamide (Baxter Pharmaceuticals, Deerfield, IL) obtained from the VA pharmacy to make a 20 mg/mL solution which was kept at 4 °C for the length of the experiment. Mice were given intraperitoneal injections of 6 μl/g body weight of this solution 24 hours prior to surgery, immediately after renal pelvis injection surgery was completed, +24 hours, and +48 hours following surgery. Controls were injected with an equal volume of normal saline. Mice received 10 μg pEF-1α-HA-m7pB and 50 μg of pTEeL by renal pelvis injection.

### Haematocrit

Mice anesthetised with isoflurane were punctured for submandibular blood collection with a 5 mm lancet (Medipoint, Inc., Mineola, NY) and blood was collected in Lithium heparin-coated microvette tubes (Sarstedt, Numbrecht, Germany). Samples were centrifuged to determine the packed cell volume and quantified in a blinded manner by the Vanderbilt University Medical Center Comparative Pathology Laboratory.

### Statistical analysis

Except in dot plots, data points represent the mean. Error bars represent the standard error of the mean. Statistics were calculated in Microsoft Excel or GraphPad Prism.

## Additional Information

**How to cite this article:** Woodard, L. E. *et al*. Kidney-specific transposon-mediated gene transfer *in vivo. Sci. Rep.* **7**, 44904; doi: 10.1038/srep44904 (2017).

**Publisher's note:** Springer Nature remains neutral with regard to jurisdictional claims in published maps and institutional affiliations.

## Supplementary Material

Supplementary Information
