# Supplementary material for: Kidney-specific transposon-mediated gene transfer in vivo
Source: Sci Rep. 2017 Mar 20;7:44904. doi: 10.1038/srep44904 (PMC5357952; doi:10.1038/srep44904)
Supplement: Supplementary Information [file srep44904-s1.pdf]

Kidney-specific transposon-mediated gene transfer *in vivo*

Lauren E. Woodard,[1,2,6] Jizhong Cheng,[6] Richard C. Welch,[2] Felisha M. Williams,[2] Wentian Luo,[2] Leslie S. Gewin,[1,2,4] and Matthew H. Wilson[1-6]

[1]Department of Veterans Affairs, Nashville, TN 37212 USA; [2]Departments of Medicine, [3]Pharmacology, and [4]Cell and Developmental Biology, Vanderbilt University Medical Center, Nashville, TN 37232 USA, [5]Department of Veterans Affairs, Houston, TX 77030 USA and [6]Department of Medicine, Baylor College of Medicine, Houston, TX 77030, USA

SUPPLEMENTARY INFORMATION

SUPPLEMENTARY FIGURES

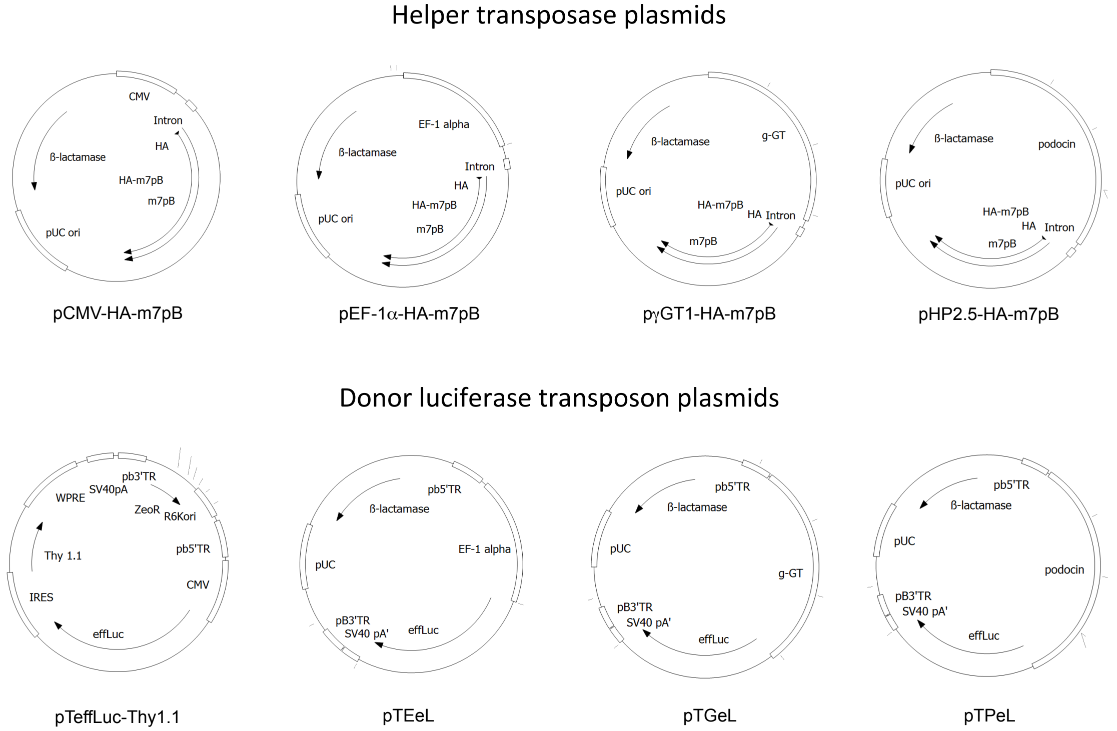

Figure S1. Maps of helper plasmids expressing hyperactive *piggyBac* transposase and donor plasmids with *piggyBac* transposons expressing luciferase. Promoters used to express either the transposase (HA-m7pB for N-terminal hemagglutinin tag, mammalian codon optimized, 7-mutation hyperactive, *piggyBac*) or effLuc (enhanced firefly luciferase) were: CMV (cytomegalovirus, constitutive viral), EF-1α (Elongation Factor-1α, constitutive endogenous), γGT1 (γ-glutamyl transferase 1; kidney tubule-specific), and podocin/HP2.5 (human podocin 2.5 kb; kidney podocyte-specific). β-lactamase: ampicillin-resistance gene; pUC ori: origin of replication; ZeoR: zeomycin resistance gene; pB3'TR: piggyBac 3' Terminal Repeat; pB5'TR: piggyBac 5' Terminal Repeat; IRES: internal ribosome entry site; Thy1.1: CD-90; WPRE: woodchuck hepatitis virus post-transcriptional regulatory element.

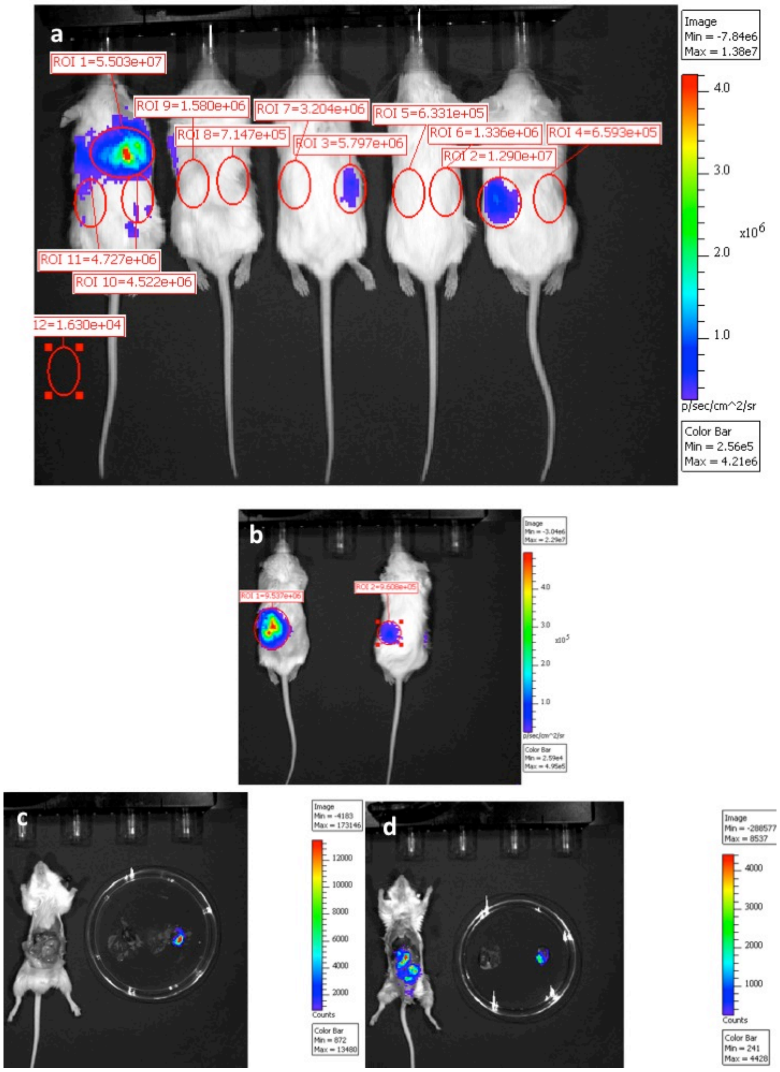

Figure S2. Comparison of organ-specific luciferase expression after hydrodynamic kidney injection of naked DNA or renal vein injection of polyethylenimine-complexed DNA. DNA-PEI complexes were made with a N/P of 6 as directed in the in vivo-jetPEI user manual (Polyplus Transfection, New York, NY).[1] Eight week old FVB male mice were sourced from the Baylor College of Medicine Transgenic Mouse Facility. (**a**) DNA-PEI complexes containing 0.140 μg/μl pTeffLucThy1.1 plasmid in 120 μl were injected into the renal pelvis with clamping of the ureter during injection (Mouse 1); 150 μl into the renal vein (Mouse 2); or 150 μl into the renal vein with clamping of vessels above the kidney during injection (Mouse 3 and Mouse 4). Mouse 5 was injected with 25 μg naked DNA in 500 μl of Mirus QR buffer solution in the renal vein. Imaging was performed one day after injections. (**b**) Mice were injected with 12.5 μg each of

pTeffLucThy1.1 and pCMV-HA-pB. The mouse on the left was given 500 µl of the DNAs diluted into Mirus QR without clamping, of which approximately 200 µl was observed leaking from the kidney. The mouse on the right was given 150 µl of DNA-PEI complex in the renal vein with a clamp upstream. Expression was one log higher in mice given hydrodynamic injection as compared with the DNA-PEI complexes one day after injections. (**c**) Dissected mouse on the left in (**b**) two days after hydrodynamic renal pelvis injection. Note the lack of expression in the peritoneum and high expression only in the injected kidney on the petri dish. (**d**) Dissected mouse on the right in (**b**) two days after DNA-PEI injection. Not only was there low expression in the kidney on the petri dish, there was ample expression throughout the peritoneum. Of note, the transfected luciferase-positive tissues from the mouse given a DNA-PEI injection had an unhealthy white appearance suggesting disruption of the lipid membrane.

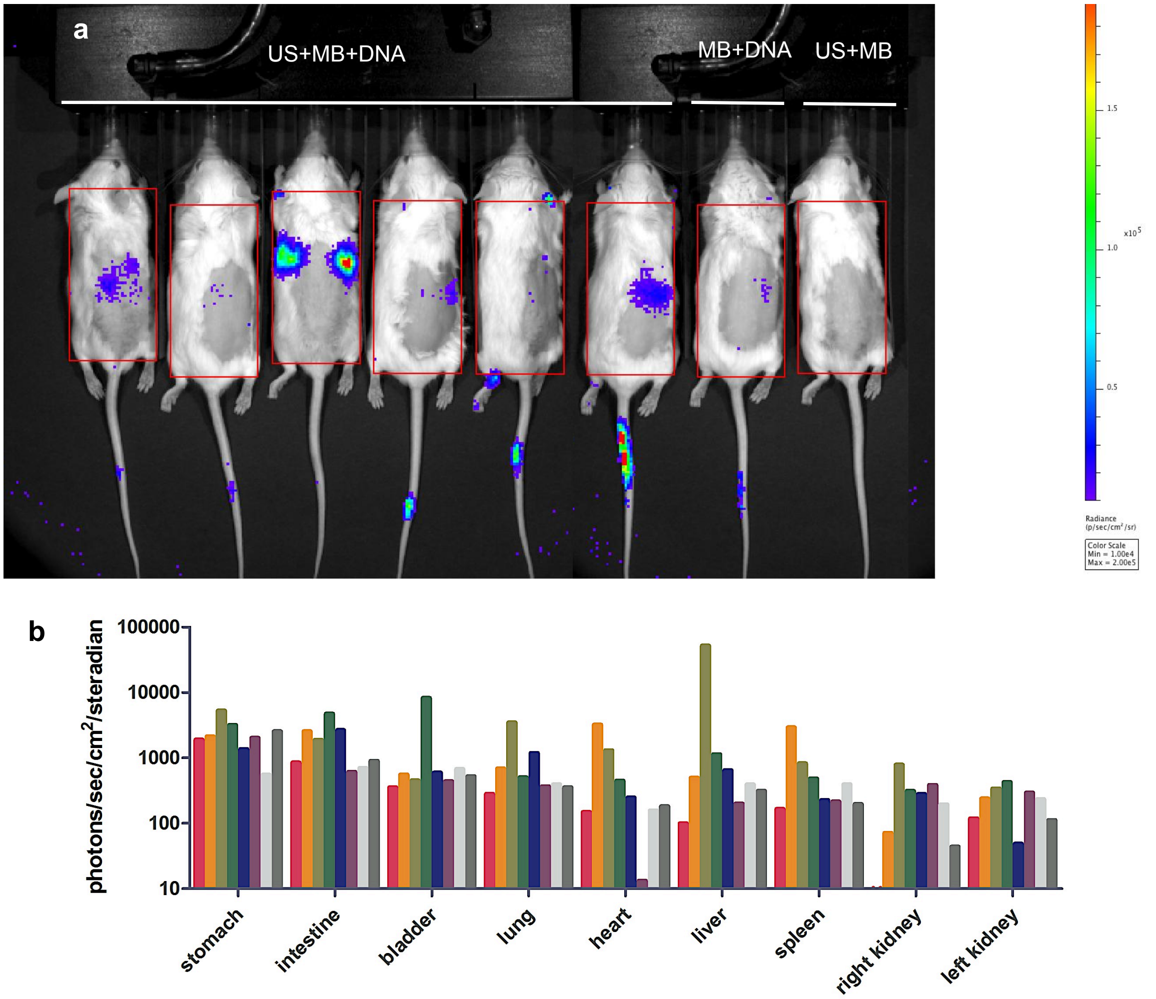

Figure S3. Luciferase expression from different organs following ultrasound microbubble-mediated transgene delivery. Mice were subjected to ultrasound microbubble transfection of the pT-effLucThy1.1 plasmid expressing luciferase from the CMV promoter. The same procedure used here for the production of the microbubbles, tail vein injection of mice, and microbubble destruction under ultrasound was previously used successfully to deliver endothelial-specific Tie2-Cre recombinase plasmid.[2] (**a**) IVIS imaging for luciferase expression one day after ultrasound microbubble treatment. Six mice received the full combination of ultrasound (US), microbubbles (MB) and 200 ug plasmid (DNA) in a volume of 300 ul . One mouse received the microbubble-complexed plasmid (MB DNA) but no ultrasound, and another mouse received microbubbles without plasmid and ultrasound (MB US). (**b**) Organ-specific expression two days after ultrasound microbubble treatment. All mice were sacrificed and their organs dissected and imaged for luciferase expression on the IVIS machine. The six mice receiving microbubble

plasmid and ultrasound (Mouse 1, red; Mouse 2, orange; Mouse 3, olive; Mouse 4, dark green; Mouse 5, blue; Mouse 6, purple) all demonstrated variable expression in different organs. The negative control without ultrasound (light grey) and negative control without plasmid (dark grey) had low expression throughout the body, while Mouse 3 (olive) showed the highest expression with highest luciferase transfection of the lung and liver. Mouse 4 (dark green) had a transfected bladder while mouse 2 (orange) showed higher expression in the heart and spleen.

REFERENCES

1. Saridey, SK, Liu, L, Doherty, JE, Kaja, A, Galvan, DL, Fletcher, BS*, et al.* (2009). PiggyBac Transposon-based Inducible Gene Expression In Vivo After Somatic Cell Gene Transfer. *Molecular Therapy* **17**: 2115-2120.
2. Huang, L, Belousova, T, Pan, JS, Du, J, Ju, H, Lu, L*, et al.* (2014). AKI after conditional and kidney-specific knockdown of stanniocalcin-1. *Journal of the American Society of Nephrology : JASN* **25**: 2303-2315.
